# Is Health Aid Reaching the Poor? Analysis of Household Data from Aid Recipient Countries

**DOI:** 10.1371/journal.pone.0084025

**Published:** 2014-01-03

**Authors:** Eran Bendavid

**Affiliations:** 1 Division of General Medical Disciplines, Stanford University, Stanford, California, United States of America; 2 Center for Health Policy and the Center for Primary Care and Outcomes Research, Stanford University, Stanford, California, United States of America; Johns Hopkins University, United States of America

## Abstract

**Objective:**

To determine the extent to which the narrowing of child mortality across wealth gradients has been related to foreign aid to the health sector in low- and middle-income countries.

**Methods:**

Mortality and wealth data on 989,901 under-5 children from 957,674 households in 49 aid recipient countries in Africa, Asia, South America, and the Caribbean between 1993 and 2012 were used in the analysis. Declines in under-5 mortality in the four poorest wealth quantiles were compared to the decline among the wealthiest at varying levels of health aid per capita using fixed effects multivariable regression models and controlling for maternal education, urbanization, and domestic spending on health among recipient countries.

**Results:**

Each additional dollar in total health aid per capita was associated with 5.7 fewer deaths per 10,000 child-years among children in the poorest relative to the wealthiest households (p<0.001). This was also true when measured in percent declines (1.90% faster decline in under-5 mortality among the poorest compared with the wealthiest with each dollar in total health aid, p = 0.008). The association was stronger when using health aid specifically for malaria than total health aid, 12.60% faster decline among the poorest compared with the wealthiest with each dollar in malaria aid, p = 0.001.

**Conclusions:**

Foreign aid to the health sector is preferentially related to reductions in under-5 mortality among the poorest compared with the wealthiest. Health aid addressing malaria, which imposes a disproportionate burden among the poor, may explain the observed effect.

## Introduction

Since the publication of the World Bank's landmark World Development Report titled *Investing in Health,* health improvements in low- and middle-income countries have been an important development agenda.[1], [2] This has been reflected in the large growth of development assistance to the health sector of developing countries (health aid), especially since 2000.[3] Nearly $200 billion dollars have been spent on health in developing countries between 2000 and 2010.[3], [4] Between 2000 and 2002 alone, donors disbursed more health aid than during the entire period from 1990 to 1999.

The relationship between increases in health aid and health outcomes has been partly characterized. The allocation of health aid does not track with disease burden or national income among recipients.[5], [6] Despite the skewed aid distribution, health aid from the US for HIV control has been associated with reductions in all-cause adult mortality, and additional mortality benefits have been attributed to health aid from The Global Fund to Fight AIDS, Tuberculosis and Malaria.[7], [8]_ENREF_7 Broadly, an analysis of health aid between 1973 and 2004 found a small contribution of health aid to reductions in child mortality, while, between 2000 and 2010, countries that received more health aid have witnessed greater increases in life expectancy and reductions in under-5 mortality.[3], [9]

While the relationships between health aid and indices of population health improvements in recipient countries are predominantly positive, the extent to which health aid has helped the poor is unknown. Poor individuals in aid recipient countries shoulder the world's highest rates of premature mortality and preventable illness.[10] Therefore, to the extent that health aid is intended to alleviate the burdens of poverty and preventable disease, donors may desire health aid to preferentially reach the poorest.

Some aspects of health aid may promote preferential increase in the supply of health care among the poor. Between 2000 and 2012, donors preferentially funded some disease areas – for example malaria, tuberculosis, and vaccine-preventable illnesses – whose burden is concentrated among the poor.[3], [11] Providing goods and services that address these diseases, then, might be expected to preferentially address disease burden among the poorest. On the other hand, the burden of HIV is not consistently concentrated among the poor, especially in sub-Saharan Africa.[12], [13] In addition, the major development assistance agencies are typically based in major urban centers.[14] If the programs funded by these agencies are also located in or near urban centers, then it is possible that health aid may not effectively reach rural, poor communities.

This analysis creates a new dataset with longitudinal data on under-5 mortality in aid recipient countries, disaggregated by wealth. This data is then used to test the extent to which health aid during the period from 1993 to 2012 modified the changes in under-5 mortality experienced by each wealth group.

## Methods

### Data Sources

All Demographic and Health Surveys (DHS) with information on household possessions for estimating wealth and birth registries for estimating child mortality were used in this analysis.[15] This yielded a total of 957,674 households and 989,901 children from 49 low- and middle-income countries with mean gross domestic product (GDP) per capita in 2010 of $1,045 (range $106–4,214 in constant 2000 US dollars).[16] Information on annual under-5 mortality rates in each wealth stratum was then matched with annual health aid disbursements to the country from the Institute for Health Metrics and Evaluation's Development Assistance for Health database.[17] The details of these procedures and the analytic approach are described below.

### Under-5 Mortality

Under-5 mortality was estimated from the DHS birth registries. Complete birth registries, obtained from women 15 to 49 years old in sampled households, contain, for every live birth, the month of birth, survival status, and age at death (in months) for children who died. Using this information, the denominator for under-5 mortality was calculated as the number of full months lived by under-5 children in every country, year, and wealth quintile during the 10-year period preceding each survey. The numerator was the number of deaths in the same population as the exposure, and under-5 mortality was the ratio of the annual number of deaths divided by the number of child-years of exposure in each country-year-wealth stratum, taking survey weights into account.

### Wealth Stratification

In DHS, wealth status is indicated in each survey using quintiles of a continuous wealth index, normalized to each survey's information. The index is obtained using a principal components analysis of household assets and services such as electricity, water supply, and floor material.[18], [19] While this index enables identification of relative wealth within surveys, it does not allow for comparisons across countries based on absolute wealth levels, since, for example, the wealth status of the poorest households in the poorest country is identical to the status of the poorest households in the least poor country.

In order to create a comparable wealth index, all 957,674 households with complete information on the following assets and services were pooled: water supply, sanitation facilities, type of flooring, electricity, the number of rooms per person living in the house, and possession of radio, television, phone (landline or cellphone), motorcycle, and car. The wealth index was then created using a principal components analysis procedure similar to the country-specific DHS approach.[19]–[21] To avoid an unbalanced influence on the index from uniquely large surveys, the index was weighted by the proportion of the national population represented by the survey. Year fixed effects were included in the principal components analysis in order to account for common changes in wealth over the period between 2003 and 2012, when the surveys were conducted. The main analysis uses quintiles of this absolute wealth index. Additional details on this procedure and the wealth index are provided in Appendix S1.

### Health Aid and Covariates

Information on health aid per capita in constant 2010 USD was obtained from the Institute for Health Metrics and Evaluation's Development Assistance for Health (DAH) database.[17] The DAH is based on the Organisation for Economic Cooperation and Development's (OECD) Creditor Reporting System (CRS).[4] The CRS tracks all-purpose development aid flows from OECD member nations. The DAH improves on the CRS by focusing on health aid, removing accounting inconsistencies, and adding information on health aid from private donors, foundations, and multilateral organizations.[22] The DAH contains total annual disbursements by donors and recipients, and allocates aid into one of six target priorities: HIV, malaria, tuberculosis, non-communicable diseases, maternal and child health, and health sector support. To test the possibility that health aid for diseases preferentially affecting poor households had relatively stronger association with under-5 mortality reductions across wealth strata, the relationship of under-5 mortality to health aid by wealth stratum was compared for total health aid and malaria health aid. If health aid was important to mortality reductions among the poor, then health aid for conditions that pose a heavy burden among the poor such as malaria was expected to have a uniquely strong relationship with mortality reductions among the poor.

In addition to health aid, several important correlates of under-5 mortality were included in this analysis. The median number of years of education of the mothers whose children were included in calculating under-5 mortality was estimated directly from the surveys for each wealth stratum. Alternative metrics of educational attainment among the mothers such as the portion who completed six years of schooling did not alter the primary findings. In addition to education, two time-varying correlates of health and development were included: recipient government spending on health per capita in constant 2010 USD, and the portion of the population living in urban areas.[16], [23] Domestic health spending per capita was used instead of GDP per capita as a surrogate of available resources for health.

### Statistical Analysis

The principal statistical models examined the association between under-5 mortality and the interaction of health aid per capita and wealth quintiles using fixed-effects (within) regressions for longitudinal data. The statistical models can be represented as:


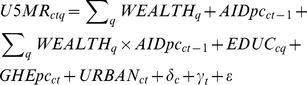


The variables in this regression represent the following measures: 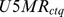 is the under-5 mortality rate in country c, year t, and wealth stratum q; 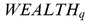 is a dummy variable representing wealth quintile q; 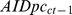 is the per-capita health aid for country c in year t-1 (lagged aid was used to allow for the delay between disbursements and any potential resultant health improvements); 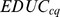 is the median number of years of education by women in country c and wealth stratum q; and 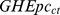, and 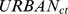 are the government health expenditures per-capita and urbanization prevalence for country c in year t. 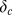 and 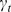
are country and year fixed effects, a series of dummy variables that control for time-invariant differences between countries and for time effects common to all countries. The coefficients on the interaction terms between health aid and wealth strata measure the relationship between under-5 mortality with increasing amounts of health aid for each wealth stratum relative to a reference wealth stratum. The wealthiest quintile was used as the reference stratum. Separate regressions were used for testing the role of total health aid and malaria-specific health aid, as described above. Robust standard errors clustered by country were used throughout to account for the serial correlation of mortality within countries.

Several robustness analyses tested the stability of the observed patterns. One analysis used log-transformed under-5 mortality rate, which relaxes the distributional assumptions on the mortality data, and in addition allows intuitive interpretations of the regression results as semi-elasticities. Additional analyses varied the lag on the health aid term from 0 to 3 years, tested the findings using a shorter recall tail on the mortality data, and examined sub-groups to test the patterns' robustness. Specifically, the primary analysis was repeated using countries with above-median GDP per capita separately from countries with below-median GDP per capita. Finally, to further examine dynamic panel data features, a model was estimated with a lagged dependent variable term on the right-hand side (similar to the model above with the addition of 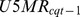 on the right hand side). The results of these robustness analyses are in Appendix S2. The analytic code is available upon request; all analyses were performed using Stata 12.1 (Statacorp).

### Ethics Statement

The study's anonymized and de-identified data were obtained with permission from the publicly available websites of MeasureDHS, OECD, the World Bank, and Institute for Health Metrics and Evaluation.[4], [15]–[17] The study was deemed Exempt by Stanford's Institutional Review Board.

## Results

**Figure 1:**
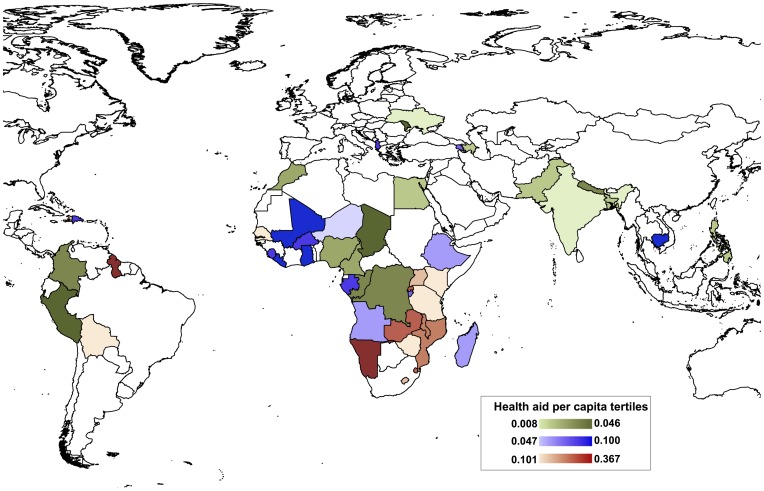
Study countries and the distribution of annual health aid per capita. Green shades represent the lowest tertile, blue shades represent the middle tertile, and red shades represent the highest tertile of average annual health aid per capita (16–17 countries in each tertile).

The study used information from all DHS surveys in the 49 study countries conducted between 2003 and 2012. Table 1 shows all the surveys used in this study, the survey dates, the number of households, and the counts of under-5 deaths and years of exposure in the decade preceding the survey. Six surveys included over 30,000 households apiece, making up 31% of all households and 26% of all under-5 person-years of exposure. Figure 1 shows the geographical distribution of the study countries and the average annual health aid per capita received by those countries during the years of observation. The 49 study countries represent 43% of the population of the 149 recipient countries in the DAH database, and received 62% of all health aid between 1990 and 2010.

**Table 1:** Study surveys, number of households, the number of under-5 deaths, and the duration of exposure.

Survey country	Survey dates	Households^1^	Number of under-5 deaths^2^	Number of under-5 child-years (1000s)^2^
Albania	10/2008–4/2009	7,987	105	24.1
Angola	1/2011–6/2011	8,023	1,275	57.5
Armenia	9/2005–11/2005	6,619	100	16.1
Armenia	10/2010–12/2010	6,665	59	13.5
Azerbaijan	7/2006–11/2006	7,124	268	24.6
Bangladesh	3/2007–8/2007	10,369	968	60.6
Bolivia	8/2003–1/2004	18,946	1,924	100.7
Bolivia	2/2008–6/2008	19,408	1,292	87.6
Burkina Faso	5/2010–12/2010	14,326	4,134	128.4
Burundi	8/2010–1/2011	8,540	1,546	58.1
Cambodia	9/2005–3/2006	14,035	1,945	84.8
Cambodia	7/2010–1/2011	15,616	1,121	76.6
Cameroon	2/2004–9/2004	10,234	2,134	64.6
Cameroon	1/2011–8/2011	14,056	2,629	95.2
Chad	7/2004–12/2004	5,321	1,996	46.9
Colombia	6/2004–7/2005	37,102	864	147.4
Colombia	11/2009–12/2010	51,184	848	185.0
Congo	7/2005–11/2005	5,822	990	37.0
Dem. Rep. of the Congo	1/2007–9/2007	8,723	2,504	67.0
Dominican Republic	3/2007–8/2007	32,228	839	117.9
Egypt	4/2005–7/2005	21,755	1,363	126.9
Egypt	3/2008–6/2008	18,866	744	102.4
Ethiopia	4/2005–8/2005	13,567	2,529	91.1
Ethiopia	12/2010–5/2011	16,509	2,633	106.0
Gabon	1/2012–5/2012	9,673	720	49.0
Ghana	9/2008–11/2008	11,660	512	26.1
Guyana	3/2009–8/2009	5,406	168	22.5
Haiti	10/2005–5/2006	7,951	1,139	54.5
Haiti	1/2012–6/2012	12,769	1,189	61.4
India	12/2005–8/2006	103,625	8,050	534.8
Kenya	4/2003–9/2003	8,476	1,169	48.1
Kenya	11/2008–3/2009	9,016	969	51.2
Lesotho	10/2009–1/2010	9,281	707	30.7
Liberia	12/2006–4/2007	6,604	1,456	47.7
Liberia	12/2008–3/2009	4,149	1,185	32.1
Madagascar	11/2008–7/2009	17,686	1,998	115.9
Malawi	1/2004–2/2005	13,457	2,979	80.4
Malawi	6/2010–10/2010	24,689	4,472	169.2
Maldives	1/2009–10/2009	6,408	202	34.4
Mali	3/2006–12/2006	12,905	5,465	115.6
Moldova	6/2005–8/2005	10,911	79	16.2
Morocco	10/2003–2/2004	10,929	714	63.7
Mozambique	8/2003–1/2004	6,876	3,261	80.1
Mozambique	6/2011–11/2011	13,268	2,014	85.1
Namibia	11/2006–3/2007	9,022	668	44.2
Nepal	2/2006–8/2006	8,660	993	57.1
Niger	1/2006–6/2006	7,624	3,288	78.4
Nigeria	3/2003–8/2003	7,055	2,301	46.9
Nigeria	6/2008–11/2008	33,900	9,047	231.0
Nigeria	10/2010–12/2010	5,871	1,542	45.2
Pakistan	9/2006–3/2007	13,463	1,677	88.1
Peru	12/2003–11/2008	39,090	1,410	181.5
Philippines	8/2008–9/2008	12,309	517	64.0
Rwanda	9/2010–4/2011	12,476	1,694	78.8
Senegal	11/2008–2/2009	9,094	3,207	124.8
Senegal	10/2010–5/2011	7,889	2,095	101.1
Sierra Leone	4/2008–8/2008	7,214	1,832	48.4
Swaziland	7/2006–3/2007	4,771	554	23.6
Tanzania	10/2004–2/2005	9,670	1,930	67.5
Tanzania	10/2007–2/2008	8,455	1,374	60.9
Tanzania	12/2009–5/2010	9,586	1,271	67.0
Timor-Leste	8/2009–2/2010	11,447	1,559	88.4
Uganda	5/2006–10/2006	8,784	2,257	68.6
Uganda	11/2009–2/2010	4,410	745	32.5
Uganda	6/2011–12/2011	8,992	1,521	66.1
Ukraine	7/2007–11/2007	13,159	51	13.5
Zambia	4/2007–10/2007	7,081	1,478	49.0
Zimbabwe	8/2005–4/2006	9,191	685	43.8
Zimbabwe	9/2010–3/2011	9,697	702	43.4
**TOTAL**		**957,674**	**117,656**	**5,453**

^1^ The number of households used is the subset of all households visited during the survey with complete information on households assets required to estimate the wealth index.

^2^ These counts represent the counts during the 10-year period leading up to the month of the survey.

**Figure 2:**
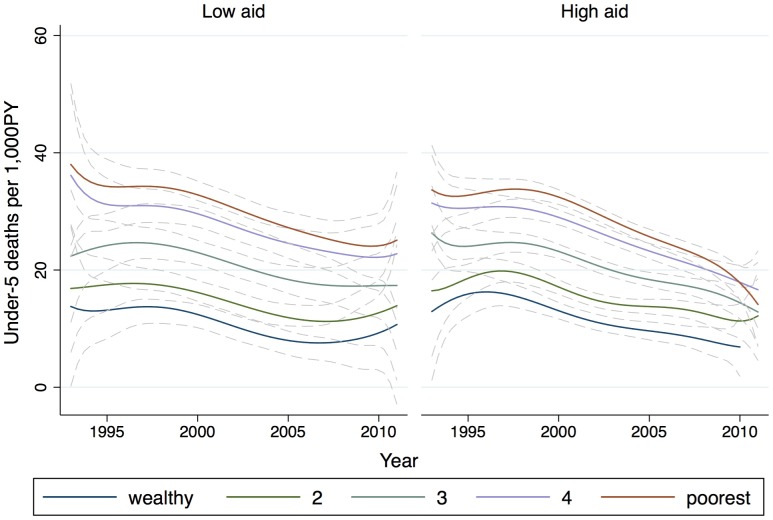
Decline in under-5 mortality by wealth stratum and health aid per capita. The lines are local polynomial smoothed fit curves (and 95% CI) with degree = 6 and an Epanechnikov kernel used for smoothing the raw annual under-5 mortality estimates for all the countries in each wealth stratum. The sub-graphs represent the countries with above-median and below-median annual total health aid per capita between 1993 and 2011. The high-aid sub-graph illustrates the convergence of under-5 mortality among the poorer quintiles, starting around the year 2000 coincident with the rapid rise of health aid.

Table 2 provides descriptive sample characteristics by wealth quintile. The Table shows that the wealth index corresponds to the possession of durable household goods and the number of rooms per person in the household. Radio is the most common asset owned by the poorest households, and nearly 100% of the wealthiest households have electricity and a refrigerator. Urban residence also increases with wealth: 6.4% of the poorest households and over 80% of the wealthiest households are located in an urban environment. Additional tests of the wealth index are provided in Appendix S1. Finally, unadjusted mortality counts show that while children in the poorest households experienced the highest under-5 mortality, they also experienced the greatest improvements in under-5 mortality, in both absolute (difference) and percentage declines (Table 2). Figure 2 illustrates this differential trend in mortality declines, and also shows that the differential declines were more prominent among the poor in countries that received more health aid.

**Table 2:** Sample description according to wealth status.

		Poorest	Less poor	Middle	Wealthier	Wealthiest
Number of households		191,583	191,489	191,657	191,417	191,528
Households possessing…(%)						
	Radio	28.7%	56.5%	61.5%	67.2%	80.7%
	Electricity	0.9%	15.4%	63.5%	96.9%	99.8%
	Flush toilets	0.4%	3.9%	26.4%	63.9%	90.1%
	Refrigerator	0.0%	0.2%	3.9%	45.7%	96.5%
	Car	0.0%	0.3%	1.7%	4.3%	29.8%
Mean no. of rooms per person (SD)		0.45 (0.24)	0.52 (0.41)	0.55 (0.46)	0.60 (0.46)	0.79 (0.63)
Living in urban areas (%)		6.4	16.9	43.6	64.1	81.1
Under-5 mortality (rate (exposure))^1^						
	1993–2000 (a)	36.9 (411.6)	34.3 (382.1)	23.2 (310.5)	13.6 (266.1)	7.9 (230.1)
	2005–2012 (b)	23.0 (492.4)	23.2 (405.1)	18.9 (309.4)	12.0 (264.1)	7.1 (198.4)
	Absolute decline (a–b)	13.9	11.1	4.3	1.6	0.8
	% decline ((a–b)/a)	37.7	32.4	18.5	11.8	10.1

^1^ Rate is the number of under-5 deaths per 1,000 under-5 person-years; exposure is the denominator. The exposure decreases with increasing wealth because of the decreasing number of children per woman.

The evidence for the interaction of health aid with wealth quintile is shown in Table 3. The coefficients on the wealth indicators represent the level differences in mortality relative to the wealthiest quintile, and the coefficients on the interaction variables represent the change in mortality for each quintile associated with different levels of health aid per capita, relative to the wealthiest quintile. The coefficient on the (lagged) aid variable stands for the naïve association of health aid with mortality among the wealthiest.

**Table 3:** Regression results of the relationship between health aid, wealth quintile, and under-5 mortality.

			Under-5 mortality	SE (p-value)	Log under-5 mortality	SE (p-value)
*Total aid*	Wealth quintile	1 - wealthiest (reference)	-	-	-	-
		2	2.59	0.97 (0.01)	29.60%	6.3 (<0.001)
		3	7.48	1.66 (<0.001)	61.10%	8.4 (<0.001)
		4	12.84	2.46 (<0.001)	83.70%	10.3 (<0.001)
		5 – poorest	14.41	3.09 (<0.001)	89.90%	11.4 (<0.001)
	Aid		0.34	0.12 (0.01)	1.50%	0.7 (0.04)
	Wealth*aid	1 - wealthiest (reference)	-	-	-	-
		2	0.01	0.09 (0.94)	−0.40%	0.7 (0.52)
		3	−0.23	0.13 (0.09)	−1.00%	0.6 (0.1)
		4	−0.51	0.2 (0.02)	−1.90%	0.7 (0.01)
		5 – poorest	−0.57	0.19 (<0.001)	−1.90%	0.7 (0.008)
	Maternal education		−0.50	0.37 (0.18)	−1.80%	0.9 (0.06)
	Gov’t health spending		0.03	0.02 (0.22)	0.00%	0.2 (0.8)
	Urbanization		0.20	0.38 (0.61)	2.10%	1.3 (0.12)
	Observations		3,199		3,110	
	R^2^		0.65		0.66	
*Malaria aid*	Wealth quintile	1 - wealthiest (reference)	-	-	-	-
		2	2.29	0.8 (0.038)	26.10%	6.1 (<0.001)
		3	6.53	1.46 (<0.001)	55.60%	8.4 (<0.001)
		4	10.86	2.13 (<0.001)	74.90%	10.2 (<0.001)
		5 – poorest	12.21	2.79 (<0.001)	79.80%	11.3 (<0.001)
	Aid		0.86	1.22 (0.48)	−1.40%	8.4 (0.87)
	Wealth*aid	1 - wealthiest (reference)	-	-	-	-
		2	−0.35	1.07 (0.74)	1.60%	8.5 (0.85)
		3	−2.86	1.02 (0.01)	−6.10%	4.2 (0.21)
		4	−4.73	1.31 (<0.001)	−13.70%	3.4 (0.01)
		5 – poorest	−5.36	1.47 (<0.001)	−12.60%	2.5 (0.001)
	Maternal education		−0.62	0.39 (0.12)	−2.30%	0.9 (0.02)
	Gov’t health spending		0.02	0.02 (0.28)	0.00%	0.1 (0.97)
	Urbanization		0.09	0.38 (0.81)	1.90%	1.3 (0.15)
	Observations		3,185		3,096	
	R^2^		0.65		0.66	

The data shows a consistent gradient where each additional dollar in health aid per capita was related to greater under-5 mortality declines among the poorer wealth quintiles. This pattern is significant when mortality declines are measured as absolute changes (0.57 fewer deaths per 1,000 person-years with each dollar in health aid among the poorest compared with the wealthiest, p<0.001), as well as when measured in terms of percent declines (1.90% greater decline in under-5 mortality with each dollar of health aid among the poorest compared with the wealthiest, p = 0.008). Within-countries, higher maternal education was associated with lower mortality, although the effect did not reach traditional statistical significance levels. Government health spending per capita was not associated with mortality changes, suggesting that, within-countries, changing levels of health spending are not important drivers of under-5 mortality in the time frame of this analysis. Among the wealthiest, higher total health aid per capita was associated with higher under-5 mortality (0.34 deaths per 1,000 child-years for every dollar in health aid, p = 0.01).

Table 3 (bottom) also shows the association between malaria-specific health aid and under-5 mortality. The data suggests that, among the three poorest wealth quintiles, malaria aid was associated with nearly 10-fold greater reductions in under-5 mortality for each dollar in aid per capita than total health aid. This finding is consistent with a role for health aid in reducing health disparities by supporting programs for disease priorities with a disproportionate burden among the poor.

Additional robustness analyses are shown in Appendix S2. The strongest association between health aid and relative mortality reduction among the poorest is found when using a 2-year lag of health aid. This is consistent with other studies that use 1−2 year lags in anticipating the impact of global health financing programs.[24], [25] Including a lagged dependent variable (the previous year's under-5 mortality rate) changed the magnitude of the effect, but did not diminish the patterns. Finally, a sub-group analysis in poorer and wealthier countries (measured by GDP per capita) yielded broadly similar patterns to the overall results (Appendix S2), suggesting that the observed pattern is less likely to be explained by patterns of convergence in mortality rates.

## Discussion

The declines in under-5 mortality in less developed countries have been welcome news, even as the drivers of this decline are incompletely characterized. This analysis presents consistent evidence from detailed person-level data that health aid has been preferentially linked to reductions in under-5 mortality among the poor. It also presents evidence consistent with an explanatory model where health aid has helped the poor when financing effective interventions for specific diseases with disproportionate burden among the poor. This is illustrated by the findings that malaria aid has been associated with a steeper gradient of mortality reductions for every dollar per capita than total health aid, and consistent with recent literature suggesting that aid-financed malaria control interventions have been associated with child mortality reductions.[26]–[28]

The 21^st^ century's largest health aid institutions – organizations like The Global Fund to Fight AIDS, Tuberculosis and Malaria, the President's Emergency Plan for AIDS Relief, the President's Malaria Initiative, and the GAVI Alliance (formerly the Global Alliance for Vaccines and Immunisation) – were typically organized to address one or a few high-burden, treatable or preventable diseases. Use of relatively modern medical and pulic health technologies such as antiretroviral therapy, insecticide-treated bed nets, and new vaccinations is a prominent feature of their implementation.[29], [30] These findings provide evidence at the population health level that the expanded availability of such technologies – financed by health aid organizations – may have promoted the observed pattern of under-5 mortality reductions.

The implications of this study are important in the current health aid climate. The economic downturn and austerity measures in donor countries, incuding the federal discretionary spending cuts in the US, led to flattening of foreign aid – and health aid – budgets.[3] Donors, then, are facing choices about the allocation of foreign aid among different priorities. If improvements in outcomes play a role in deciding on aid sector allocation, then this study's findings suggest that health aid may be a desirable investment; the evidence on the effectiveness and equity of non-health aid (such as economic assistance) is more equivocal.[31] In addition, the flat resource ceiling in the health aid sector is fueling discussions of health aid priorities.[32] The Global Fund to Fight AIDS, Tuberculosis and Malaria, for example, is shifting its core priorities to include greater emphasis for health sector support. The Paris Declaration on Aid Effectiveness, in addition, asks donors to align and harmonize their investments with recipient priorities.[33] This study suggests that shifting donor priorities away from current infectious disease control priorities may have unintended consequences, especially on the poor. For example, increasing health aid for non-communicable diseases at the cost of decreasing commitments for existing priorities such as malaria control could preferentially benefit the wealthier, for whom non-communicable diseases pose a significant burden, and harm the poor. Historically, withdrawal of support for malaria control programs has led to unintended resurgence of malaria and worsening child mortality.[34]

This study's findings come with caveats of observational studies that deserve explicit consideration. One alternative explanation for the observed findings is that health aid was targeted to countries with large poor populations, and the association merely reflects the rapid but unrelated declines in under-5 mortality in those countries over the study period. A related argument is that health disparities across countries are converging for reasons that are unrelated to health aid such as economic growth. However, the level of economic development does not explain the observed effect, and the patterns in low-aid recipient countries suggest a different relationship between aid and mortality improvements than in high-aid countries. The other important limitation is that wealth status is only measured at the time of the survey. In order to lower the possibility of large shifts across wealth strata, durable goods that reflect long-term wealth were emphasized in the set of household possessions used in the calculation of the wealth index.

This analysis also shows that health aid wasrelated to higher under-5 mortality among the wealthiest. It is possible that health aid crowded out health services that are uniquely important for health among the wealthiest.[35] More plausibly, this may reflect the effect of HIV. Several countries that received substantial amounts of health aid also have heavy HIV burden that is highest among the wealthiest. Since HIV plays an important role in under-5 mortality trends in countries such as Swaziland and Zimbabwe, it would appear that health aid was related to higher under-5 mortality among the wealthiest.[36]

In summary, this study suggests that the past decade's financing priorities have been associated with greater improvements in under-5 mortality among the poor compared with the wealthier in aid-recipient countries. These findings could help in informing aid allocation decisions (when prioritizing pro-poor aid sectors) as well as decisions within the health aid community. In deciding on future health aid priorities, this study provides the first analysis of the aid-related factors involved in promoting equitable health improvements.

## Supporting Information

Appendix S1 Wealth Index construction and validation.
(DOCX)

Appendix S2 Robustness analyses.
(DOCX)
