# Supplementary material for: Is Health Aid Reaching the Poor? Analysis of Household Data from Aid Recipient Countries
Source: PLoS One. 2014 Jan 3;9(1):e84025. doi: 10.1371/journal.pone.0084025 (PMC3880283; doi:10.1371/journal.pone.0084025)
Supplement: Appendix S1 — Wealth Index construction and validation. (DOCX) [file pone.0084025.s001.docx]

SUPPLEMENTARY APPENDIX S1 FOR “IS HEALTH AID REACHING THE POOR? ANALYSIS OF HOUSEHOLD DATA FROM AID RECIPIENT COUNTRIES”

The Supplementary Appendix contains information on the construction and validation of the wealth index

# Creation and validation of a cross-country wealth index

## Background and procedure for creating the wealth index

Use of household data from DHS surveys for creating a wealth index has been discussed extensively in the literature.[^1^](#_ENREF_1)^,^ [^2^](#_ENREF_2) The use of household possessions is used instead of consumption, income, or expenditures. It is generally thought that wealth index represents a more permanent status than consumption or income. More complete discussions of the advantages and tradeoffs involved in creating a wealth index are presented elsewhere.[^1^](#_ENREF_1)^,^ [^3^](#_ENREF_3) This study follows the general procedure of the DHS in using principal components analysis to generate an index from a set of household durable goods and services. The set of assets was modified for this study for two reasons: to reduce the reliance on non-durable goods such as clothing and jewelry; and to reflect a set of assets that is uniformly collected in all countries. The possible downside of limiting the set of assets is reduced discriminating ability; however, as shown below, this concern did not present a serious challenge.

The following goods and services were used in the creation of the wealth index:

| Electricity (Y/N) | Radio (Y/N) |
| --- | --- |
| Television (Y/N) | Refrigerator (Y/N) |
| Motorcycle (Y/N) | Car (Y/N) |
| Phone (landline or cell, Y/N) |  |

Number of rooms per person living in the house (continuous)

Type of flooring (5-point scale)

Water source (5-point scale)

Sanitation facilities (5-point scale)

The classification scheme for water source and sanitation services followed the scheme used in Gunther and Fink’s analysis of 172 DHS surveys.[^4^](#_ENREF_4) The scheme is available from the author. Flooring classification used the following scheme:

| Floor type | Quality |
| --- | --- |
| Mud/clay/earth | 1 |
| Sand | 1 |
| Dung | 2 |
| Brick | 3 |
| Carpet | 3 |
| Raw wood planks | 3 |
| Palm, bamboo | 4 |
| Vinyl, asphalt strips | 4 |
| Cement | 4 |
| Polished stone/marble/granite | 4 |
| Parquet, polished wood | 5 |
| Ceramic tiles | 5 |

These assets were then used in a principal components analysis (PCA). The analysis was performed on the entire set of pooled 957,674 households from 69 DHS surveys. Survey size varied from around 4,000 to over 100,000. This could lead to unbalanced influence of the large surveys. To relax this concern, a frequency weight was constructed from the ratio of the country population divided by the size of the survey. In addition, to allow for the variation in the timing of the survey between 2003 and 2011, we used year fixed effects in the PCA. The first principal component was then used to predict a uni-dimensional index. The first component loadings are shown below:

| Component | Loading |
| --- | --- |
| Water_qual | 0.5488 |
| Toilet_qual | 0.7095 |
| Floor_qual | 0.7414 |
| Electricity | 0.7327 |
| Radio | 0.352 |
| TV | 0.8073 |
| Fridge | 0.7511 |
| Motorbike | 0.3896 |
| Car | 0.39 |
| Phone | 0.6938 |
| Rooms_pp | 0.2159 |

The population was then divided into quantiles (5 quintiles for the primary analysis) based on the value of the index.

## Wealth index validation

In addition to ascertaining an adequate distribution of the wealth index, I performed two assessments to examine the extent to which the wealth index reflected a meaningful correlate of health and socioeconomic status. First, the new index was correlated with rates of stunting and underweight among all under-5 children in the household (defined as z-score<=-2SD relative to a standard reference population). Second, the index was correlated with levels of education of the head of the household. (Neither education or child nutritional status were used in the construction of the index.) I expected a consistent gradient for both measures, even as this index was uniformly applied to 49 countries. The following figures show the associations of the wealth index with child nutritional status and education.

Figure A1a: the portion of the population in the wealth decile that is stunted, defined as height-for-age less than or equal to 2SD below the median of a reference population. The gradient is monotonic in the wealth index for a population of 436,882 children from the 49 study countries.

Figure A1b: the portion of the population in the wealth decile that is underweight, defined as weight-for-age less than or equal to 2SD below the median of a reference population. Together with stunting, this graph illustrates a close association between child nutritional status and the wealth index.

Figure A2: the portion of households where the head of the household completed at least 12 years of school out of 918,501 households with information on the head of household’s education.

**Reference**

1. Rutstein SO, Johnson K. The DHS wealth index: ORC Macro, MEASURE DHS; 2004.

2. Filmer D, Pritchett LH. Estimating Wealth Effects Without Expenditure Data—Or Tears: An Application To Educational Enrollments In States Of India*. *Demography* 2001; **38**(1): 115-32.

3. Rutstein SO. The DHS wealth index: approaches for rural and urban areas. *Washington, DC: Macro International Inc* 2008.

4. Günther I, Fink G. Water, Sanitation and Children’s Health. *Prospects* 2010.
