# Supplementary material for: Is Health Aid Reaching the Poor? Analysis of Household Data from Aid Recipient Countries
Source: PLoS One. 2014 Jan 3;9(1):e84025. doi: 10.1371/journal.pone.0084025 (PMC3880283; doi:10.1371/journal.pone.0084025)
Supplement: Appendix S2 — Robustness analyses. (DOCX) [file pone.0084025.s002.docx]

SUPPLEMENTARY APPENDIX S2 FOR “IS HEALTH AID REACHING THE POOR? ANALYSIS OF HOUSEHOLD DATA FROM AID RECIPIENT COUNTRIES”

The Supplementary Appendix contains the following robustness analyses:

Effect of alternative lag assumptions on health aid

Effect of shorter recall window for mortality estimation

Subgroup analysis using low and high GDP country groups

Alternative model specifications using lagged dependent variable

# Robustness Analyses

## Effect of alternative lag assumptions on health aid

A lag of 1-year was selected for the primary analysis. This lag reflects a delay between aid disbursement and any anticipated health benefits. A lag of 1-2 years has been used in other studies examining the effectiveness of health aid organizations. The table below displays the coefficients (and p-values) on the aid and aid * wealth interactions using lags of 0-3 years using the untransformed under-5 mortality outcomes.

|  | Lags | 0 years | 1 (main analysis) | 2 years | 3 years |
| --- | --- | --- | --- | --- | --- |
| Aid |  | 0.34** | 0.34* | 0.43** | 0.33** |
| Wealth*aid | 1 - wealthiest (reference) | - | - | - | - |
|  | 2 | 0.00 | 0.01 | 0.43 | 0.07 |
|  | 3 | -0.20 | -0.23 | -0.04 | -0.22 |
|  | 4 | -0.49* | -0.51* | -0.29* | -0.53 |
|  | 5 – poorest | -0.54** | -0.57** | -0.60** | -0.64* |
| Obs |  | 3,199 | 3,185 | 2,915 | 2,626 |
| R^2^ |  | 0.65 | 0.65 | 0.66 | 0.65 |

* p≤0.05 ** p≤0.01

## Effect of shorter recall window for mortality estimation

Shortening the recall window for the mortality estimations – the period of time prior to the survey used to estimate under-5 exposure and mortality – has two main benefits. First, it reduces the effects of recall bias due to imperfect recall of dates of birth and ages at death of events farther in the past. In addition, it reduces the misidentification of wealth stratum by nudging the window of analysis closer to the date of the survey. The following table shows the primary specification with untransformed under-5 mortality and 1-year lagged health aid, where the outcome data is calculated using 9, 8, 7, 6, and 5 years prior to the date of the survey. The table shows that while shorter recall windows resulted in fewer observations and smaller R^2^, the direction and magnitude of the effect remained mostly unaffected.

|  | Recall window (years) | 9 | 8 | 7 | 6 | 5 |
| --- | --- | --- | --- | --- | --- | --- |
| Aid |  | 0.30(0.009) | 0.24(0.02) | 0.21(0.05) | 0.19(0.09) | 0.17(0.18) |
| Wealth*aid | 1 - wealthiest (reference) | - | - | - | - | - |
|  | 2 | -0.02(0.86) | -0.03(0.78) | -0.05(0.60) | 0.01(0.92) | -0.03(0.81) |
|  | 3 | -0.29(0.03) | -0.23(0.04) | -0.29(0.02) | -0.22(0.05) | -0.22(0.06) |
|  | 4 | -0.42(0.03) | -0.36(0.04) | -0.37(0.03) | -0.31(0.04) | -0.26(0.06) |
|  | 5 – poorest | -0.60(0.001) | -0.56(0.001) | -0.52(0.003) | -0.40(0.02) | -0.34(0.02) |
| Obs |  | 2,934 | 2,643 | 2,358 | 2,065 | 1,768 |
| R^2^ |  | 0.64 | 0.64 | 0.62 | 0.61 | 0.58 |

## Subgroup analysis using low and high GDP country groups

This analysis examined whether the patterns of preferential mortality reduction persisted in country subgroups. We tested the main specification among countries with GDP per capita above and below the median in 2010. The table below shows the results using untransformed under-5 mortality and 1-year lag on health aid. While the associations are weaker in wealthier countries, the trends are consistent, with the poorest quintile experiencing greater declines in under-5 mortality with increasing health aid relative to the wealthiest quintile.

|  | Country group | Below median GDPpc | Above median GDPpc |
| --- | --- | --- | --- |
| Aid |  | 0.59(0.07) | 0.10(0.24) |
| Wealth*aid | 1 - wealthiest (reference) | - | - |
|  | 2 | -0.53(0.02) | 0.13(0.14) |
|  | 3 | -0.80(0.008) | 0.04(0.71) |
|  | 4 | -0.95(0.001) | -0.15(0.34) |
|  | 5 – poorest | -0.99(<0.0001) | -0.24(0.16) |
| Obs |  | 1,785 | 1,414 |
| R^2^ |  | 0.64 | 0.60 |

## Alternative model specifications using lagged dependent variable

Dynamic panel considerations, where current values of the dependent variable are influenced by past values and in addition have a dynamic relationship with the independent variables are important to consider in this context. While an extensive literature exists on the topic, the application in practice is often limited by uncertainty about the relationship of lags to the current values.[^1^](#_ENREF_1)^,^ [^2^](#_ENREF_2) In a simplified attempt that can reasonably thought to reduce bias in the estimates, a model specification with a 1-year lag on the dependent variable was tested. The coefficient on interaction term between the poorest quintile and 1-year lagged health aid decreased from -0.57 to -0.47, p<0.001, while the R-squared increased to 0.72.

1. Roodman D. Doubts about the evidence that foreign aid for health is displaced into non-health uses. *The Lancet* 2012; **380**(9846): 972-3.

2. Arellano M, Bond S. Some tests of specification for panel data: Monte Carlo evidence and an application to employment equations. *The Review of Economic Studies* 1991; **58**(2): 277-97.
